# Study on Supramolecules in Traditional Chinese Medicine Decoction

**DOI:** 10.3390/molecules27103268

**Published:** 2022-05-19

**Authors:** Yuan Gao, Yingying Dong, Qin Guo, Huanhuan Wang, Mei Feng, Zhengshen Yan, Dong Bai

**Affiliations:** 1 Institute of Basic Theory for Chinese Medicine, China Academy of Chinese Medical Sciences, Beijing 100700, China; gaoyuan_0714@163.com (Y.G.); 17862997138@163.com (Y.D.); gqyuanzhi@163.com (Q.G.); juliuck@163.com (Z.Y.); 2 Basic Medical School, Shanxi University of Chinese Medicine, Xianyang 712046, China; 15670625429@163.com (H.W.); fengmei259@163.com (M.F.)

**Keywords:** decoction, supramolecules, self-assembly

## Abstract

With the application of the concept of supramolecular chemistry to various fields, a large number of supramolecules have been discovered. The chemical components of traditional Chinese medicine have various sources and unique structures. During the high-temperature boiling process, various active components form supramolecules due to complex interactions. The supramolecular structure in a traditional Chinese medicine decoction can not only be used as a drug carrier to promote the absorption and distribution of medicinal components but may also have biological activities superior to those of single active ingredients or their physical mixtures. By summarizing the relevant research results over recent years, this paper introduces the research progress regarding supramolecules in various decoctions, laying a foundation for further research into supramolecules in traditional Chinese medicine decoctions, and provides a new perspective for revealing the compatibility mechanisms of traditional Chinese medicine, guiding clinical medications, and developing new nanometers materials.

## 1. Introduction

Jean-Marie Lehn, winner of the Nobel Prize in Chemistry, proposed the concept of supramolecular chemistry in 1973, which is a chemistry that studies supramolecule aggregates formed by the self-assembly of two or more molecules due to intermolecular non-covalent bonding forces [1]. As this concept gradually became known, researchers discovered many supramolecules in the soup. These self-assembled structures are composed of natural ingredients in food and formed by heat induction during boiling. Fish soup is nutritious and popular. When using an optical microscope and a laser scanning confocal microscope to observe tuna fish head soup, it was found that there were many different-size spherical supramolecules in the soup. The main structure was formed by triglycerides, and the phospholipids were distributed on the surface of the spherical structure. Carbohydrates were embedded in the periphery of the structure. These supramolecules have anti-oxidative ability in vitro, being also able to scavenge intracellular free radicals [2]. When further studying the influencing factors for the formation of supramolecules in soup, it was found that the addition of salt could not only adjust the taste and add umami, and promote the dissolution of nutrients and taste substances, but also increase the capacity for binding between protein and fat, enabling the protein to better encapsulate the fat and promote the formation of supramolecules [3,4]. In addition to fish soup, pig bone soup has also been separated and purified to obtain supramolecules, which are mainly composed of fat and protein, and they exhibited intracellular ROS scavenging activities and modulated the cellular membrane potential of mucosal macrophages via direct interaction [5]. Wang analyzed supramolecular nanoparticles isolated from the freshwater clam (*Corbicula fluminea* Muller) soup. The food-derived nanoparticles consisted of polysaccharides, lipids and proteins, carried six kinds of phytosterols occupying over 80% of total phytosterol content of the soup. Based on the results of characterization and identification, the structure of the supramolecular nanoparticles was deduced as polysaccharides assembling the outer layer of particles with lipids bound to the surface, while proteins were hidden inside the particles. Studies have found in a variety of models that the supramolecular nanoparticles effectively inhibited cholesterol uptake and have the effects of treating non-alcoholic fatty liver, which resonates with the traditional belief that freshwater clam soup provides hepatoprotective benefits [6,7,8]. From these studies, it can be observed that many supramolecules are formed during the cooking of food, and these structures are mainly composed of nutrients and have certain medicinal effects.

The processing method for soup can easily remind us of Chinese medicine decoction. Similar to with soup, water is used as a solvent to extract various components of Chinese medicine under boiling. The water-soluble components of an herbal medicine can be dispersed in the decoction in a molecular state, the fat-soluble components will be in an emulsified state, and some insoluble components will exist in the state of particles. On the other hand, complex reactions occur between the ingredients during the process of boiling, forming colloidal particles, suspensions, etc. The complexity of the active ingredients in compound decoctions of traditional Chinese medicine is very similar to that of the nutritional ingredients in food. Research on food shows that cooking soup is not a simple hot-water-extraction process. Accompanied by the ingredients in the food migrating from cells to hot water, various components spontaneously form stable and orderly supramolecules [9]. Decoction is one of the most commonly used dosage forms in the clinical application of traditional Chinese medicine. When the ingredients in an herbal medicine are released into water by boiling, whether the effective ingredients also form supramolecules in the decoction and whether the formed supramolecules have medicinal effects are of interest. These issues are worthy of in-depth study. By combining the concept of supramolecular chemistry with the theory of traditional Chinese medicine, it can be observed that the colloidal particles and suspensions in a decoction are supramolecules formed by the self-assembly of the components of traditional Chinese medicine under non-covalent forces such as electrostatic attraction, van der Waals forces, hydrophobic forces, and hydrogen bonds. To date, supramolecular structures have been found in many decoctions [10]. The study of supramolecules in decoctions can not only clarify the mechanism of the self-assembly of supramolecules on a material basis but may also provide a new perspective for clarifying the material basis of the pharmacodynamics in a decoction.

## 2. Research on Supramolecules

### 2.1. The Essence of Supramolecules

When studying the chemical composition of *Poria cocos*, Zhi [11] found that the *Poria cocos* crude extract became viscous after cooling and even solidified to form a “jelly-like” substance. The same phenomenon also occurs after removing the solvent from a sample obtained after the column chromatography of the crude extract of *Poria cocos*. Therefore, they studied 63 crude extracts of traditional Chinese medicine and found that 16 crude extracts had the ability to form “jelly-like” substances. These are actually supramolecular gel states formed by the components in the decoction through self-assembly. In a supramolecular system, various specific structures are formed between molecules through electrostatic attraction, van der Waals forces, hydrophobic forces, and hydrogen bonding. The products can be expressed as nanoparticles, liposomes, nanotubes, vesicles, precipitation, etc. [12]. After the supramolecules are formed, each component still maintains some of its unique physical and chemical properties and, at the same time, exhibits a special overall function due to the interaction with each other [13].

**Figure 1:**
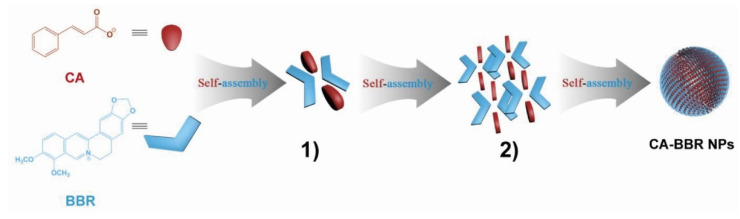
Schematic representation of the self-assembly mechanism of the cinnamic acid–berberine supramolecules (CA–BBR NPs). (**1**) One-dimensional self-assembled unit of CA–BBR supramolecules. (**2**) Three-dimensional packing structure of CA–BBR supramolecules [15].

Studies have shown that the supramolecules formed by self-assembly are a spontaneous, tight, orderly combination of several components. The supramolecules in the Huanglian Jiedu decoction are composed of baicalin and berberine in a molar ratio of 1:1 and formed through electrostatic attraction to form a complex molecule with an amphiphilic structure. This complex has a hydrophilic sugar ring at one end and two hydrophobic cores at the other end; the amphiphilic complex serves as the basic unit for further self-assembly to form supramolecules [14]. When the supramolecules formed during the co-decoction of *Coptidis rhizoma* and *Cinnamomi cortex* were studied, Huang [15] found that the active ingredients berberine (BBR) and cinnamic acid (CA) could spontaneously form a butterfly-shaped one-dimensional self-assembled unit under the action of hydrogen bonding and π–π stacking, as shown in Figure 1. The continuous accumulation of one-dimensional units finally formed three-dimensional supramolecules with good antibacterial activity.

**Figure 2:**
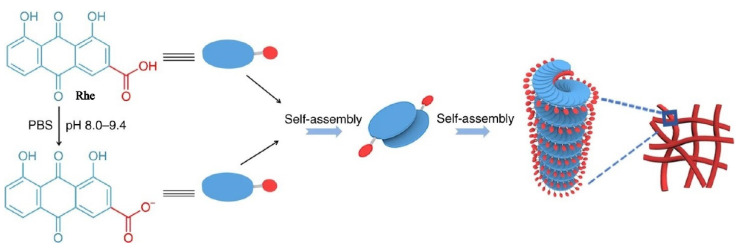
Schematic representation of the self-assembly mechanism of the rhein hydrogel [16].

Zheng [16] found that certain rhein was deprotonated to form rhein sodium salt when the pH value was between 8.0 and 9.4. As shown in Figure 2, Rhein monomer and rhein sodium salt were arranged in a J-type aggregation manner by π–π stacking and hydrogel bonding to form a dimer. Due to the electrostatic repulsion between the carboxylic acid ions, two molecules tended to be arranged in opposite direction. The rhein molecules continually added to the per-existing aggregates in a left helix fashion, resulting in the formation of the nanofiber with a left-handed helical configuration. The nanofibers further crosslinked to form three-dimensional networks, which showing excellent stability, sustained release, and thixotropy. More importantly, the experiments showed that the rhein hydrogel exhibited better neuroinflammatory prevention effect than the free drug due to the sustained release control of the nanofibers.

**Figure 3:**
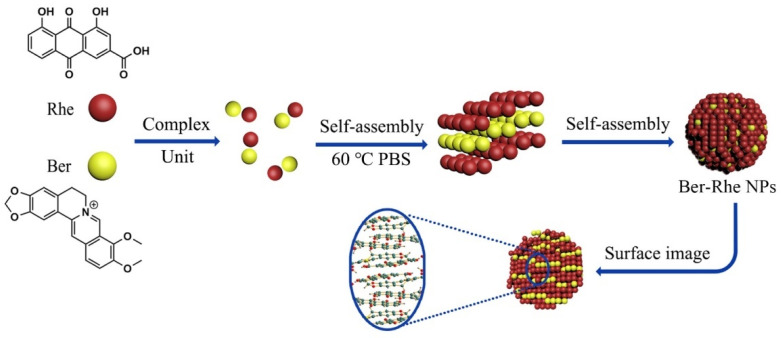
Schematic representation of the self-assembly mechanism of the berberine–rhein (Ber–Rhe) supramolecules [17].

When studying the compatibility of *Coptis chinensis* and *Rheum palmatum*, it was found that rhein forms a layered framework under the action of hydrogen bonds, and berberine is embedded in it through π–π interactions and electrostatic forces to form a stable, layered three-dimensional structure, as shown in Figure 3. Compared to the free drug, the antibacterial effect of berberine was obviously enhanced by self-assembling into nanoparticles with rhein. In the efficacy experiments, untreated bacteria exhibited a smooth surface; the nucleoid of the cell center was intact. The membrane of bacterial exhibited shrunk and fusion with massive cytoplasmic leakage after incubation with berberine–rhein supramolecules, the morphology of the bacteria was severely altered and led to death. Rhein has multiple alternating phenolic hydroxyl, carbonyls, and carboxyl groups, which can form hydrogen bonds with amide and carboxyl groups at the peptidoglycan terminal on the cytomembrane of the bacteria. When bacteria incubation with berberine–rhein supramolecules, Scientists inferred that plenty of supramolecules were adhered to the surface of the bacteria; local high concentration of berberine and rhein would damage the membrane ion channel protein and lyse the bacteria. Therefore the synergistic antibacterial effect of berberine and rhein made the nanoparticles more effective [17]. It can be observed from these studies that the process of self-assembly is not a simple superposition of weak forces among a large number of atoms, ions, and molecules but a complex synergy. The non-covalent bond between molecules is the core force that drives the formation of supramolecules. The formed supramolecule is a spontaneous combination of several components from traditional Chinese medicine decoction forming a tight and orderly whole. These supramolecular structures have some biophysical and biochemical properties, which are summarized in Table 1.

However, it should be noted that the ordered structures of supramolecules are not fixed. Under different conditions, the structures of supramolecules and the responses to environmental changes (such as pH) change, and the efficacy of a drug based on them will not be the same. The real-time monitoring of the boiling process for the Maxing Shigan decoction showed that the mixing degree for the particles decreased during the process, the particle-size distribution narrowed and finally stabilized at approximately 170 nm, and the organic and inorganic components were non-uniformly distributed among the phases. Studies have shown that the forces between molecules are mainly hydrophobic interactions and hydrogen bonding. Breaking these forces leads to changes in the supramolecular structure and a decrease in antibacterial activity in vivo and vitro [34]. The supramolecular nanoparticles in the *Cordyceps militaris* decoction system showed a pH response, and the supramolecules have the ability to embed and release molecules with a change in pH [35]. Research into the *Puerariae thomsonii* Radix Aqueous decoction showed that the size of the supramolecules depended on the concentration of the active ingredient, and the smaller the supramolecule’s diameter, the better the drug absorption effect [18]. As a type of supramolecule, the saikosaponin a isolated from *Radix bupleuri* can form a gel in water, methanol–water, ethanol–water, and acetone–water, but scanning electron microscope (SEM) results showed that the gel in the different solvents had different appearances [36]. The orderliness of the supramolecules showed that the research of the supramolecules in the decoction was reproducible. Scientists can analyze the contents of active ingredients in supramolecules and use monomer components to simulate supramolecules with a clear material basis and more practical value. The influence of different decoction conditions on the structure and function of supramolecules is also an important condition that cannot be ignored in the process of developing and synthesizing supramolecules.

### 2.2. Separation Method for the Supramolecules

The selection of the separation conditions for supramolecules is an important prerequisite for research. Dialysis involves the diffusion of substances with molecular weights less than a defined value out of a dialysis bag, removing the true-solution phase in the decoction. Centrifugation uses the centrifugal force generated by the high-speed rotation of a centrifuge rotor to accelerate the sedimentation of the particles in the liquid and separate substances of different molecular weights in the system.

In addition, a multi-angle light scatter detector (MALS) can also separate the supramolecules in a decoction from other components or separate structures with different particle sizes or different charging properties. The most commonly used method is the combination of gel chromatography and MALS. The separation principle of gel chromatography is the molecular-sieve effect. When a sample solution containing various molecules flows slowly through a gel-chromatography column, the macromolecular substances are difficult to enter the micropores of the gel particles due to their large diameters, so they move down faster during elution. In addition to diffusing in the interstices of the gel particles, small molecules can also enter the micropores of the gel particles, so the movement is relatively slow. Therefore, substances with different molecular weights are separated; then, UV detectors and MALS are used to continuously monitor the eluent. The molar mass and root mean square (RMS) radius can be analyzed, and the target supramolecules can be collected.

### 2.3. Characterization Method for Supramolecules

Due to the lack of technologies and methods for observing nanostructures in the past, research on supramolecules in decoctions has been progressing slowly. In recent years, with the popularization of various analytical methods, such as dynamic light scattering (DLS) and transmission electron microscopy (TEM), supramolecules in decoctions have gradually been discovered. The polymer dispersibility index (PDI), element composition, and morphology of supramolecules and particle size have gained much attention. Researchers currently use DLS to measure the size distributions and zeta potentials of supramolecules and use nanoparticle analyzers to determine the particle sizes of supramolecules. One of the most intuitive characterization methods is the use of TEM to observe the morphology and dispersion of supramolecules and to measure their sizes. The principle of TEM is projecting an accelerated and concentrated electron beam onto a very thin sample. Electrons change direction due to collisions with atoms in the sample, thereby producing solid angle scattering. The size of the scattering angle is related to the density and thickness of the sample, so images with a different brightness and darkness can be formed.

Much information regarding supramolecules can be understood through these characterization methods. For example, atomic force microscopy was used to study the morphology and structure of polysaccharides extracted from the root cortex of *Paeonia suffruticosa*. The results showed that the polysaccharide molecules had a nearly spherical structure, and the diameter was 50–80 nm; they were presumed to be the basic structural unit of polysaccharides. Under certain concentrations and conditions, the small particles aggregated to form a hollow sphere structure with a diameter of approximately 170–220 nm. Heating the polysaccharide solution to 80 °C could promote the self-assembly of polysaccharide molecules, which could form a long-chain supramolecular structure through inter-chain hydrogen bonding. Each subunit in this supramolecule was formed by the agglomeration and compaction of small spherical particles [37]. These structural characterization methods are very helpful for understanding the form and formation of supramolecules.

### 2.4. Structural Analysis Method for the Supramolecular Structure

When the characterization of the supramolecules was completed, researchers tried to clarify the specific structural information of the supramolecules. The formation of a fingerprint can be used to compare the changes in the ingredients in the decoction before and after the formation of the supramolecules so as to clarify the relationship between the formation of the supramolecules and the ingredients. Through the study of the spectral results of the supramolecules, it is possible to speculate on the interaction between the components, such as comparing the Ultraviolet−visible spectroscopy, Fourier transforms infrared spectroscopy, and fluorescence emission spectra of the supramolecules and each monomer, which can reveal the relationship among the components. 

In order to further confirm the types and positions of the interactions between the components, the high-resolution mass spectroscopy of the supramolecules can be measured, and then, the chemical shift with the monomer components under the same conditions can be compared and the position of the action can be clarified. 1H NMR and ROESY 2D NMR spectra were used to ensure the formation and conformation of the complex units as well as the self-assembly mechanism. The continuous progress of methods for obtaining supramolecular structural information has greatly facilitated further study of supramolecules in traditional Chinese medicine.

## 3. Study of the Supramolecules of Traditional Chinese Medicine Decoctions

### 3.1. Research on the Supramolecules in the Prescription Decoctions

#### 3.1.1. Baihu Decoction

The Baihu decoction is made from *Anemarrhena asphodeloides*, *Glycyrrhizae*, *Japonica rice*, and *Gypsum*. Lv [38] used high-speed centrifugation and dialysis technology for the phase splitting of the Baihu decoction. Then, they used HPLC to determine the contents of the effective ingredients in the Baihu decoction in different phases. The results showed that the main components of each phase of the Baihu decoction were basically the same, and the content of active ingredients in the nanophase was significantly higher than that in the other phases. Therefore, it is speculated that the supramolecules in the nanophase of the Baihu decoction have a solubilizing effect on the main antipyretic components. In order to study the mechanism of the formation of the supramolecules in the Baihu decoction, particle size, salinity, conductivity, surface tension, TEM, and fingerprint of the supramolecules were measured. Based on the results, it is speculated that the four traditional Chinese medicines in the Baihu decoction all played important roles in the formation of the supramolecules. The macromolecules, such as proteins and polysaccharides, produced by the boiling of *Japonica rice* form particles with pores that can serve as the main structure of the supramolecules, allowing the chemical components in the decoction to be embedded. *Anemarrhena asphodeloides* contain many antipyretic-related medicinal ingredients, such as neomangiferin and mangiferin, which are poorly water-soluble. *Gypsum* contains many inorganic ions. On the one hand, Fe^2+^ and Fe^3+^ form iron oxides or iron hydroxides with high surface energy that can serve as the central cores of supramolecules. The insoluble components attracting by cores are highly enriched at the periphery of the core, thereby exerting a solubilization effect. On the other hand, Ca^2+^, Mg^2+^, and Zn^2+^ act as zeta-potential regulators that are adsorbed in the supramolecules to regulate the stability of the structure. There are many saponins in *Glycyrrhizae*, such as glycyrrhizic acid and glycyrrhetinic acid, which are important surface-active substances that can improve the solubility of mangiferin and neomangiferin and regulate the stability of the supramolecules [19,39]. An efficacy test showed that the antipyretic effect on rabbits and the effect of reducing the level of inflammatory factors in the serum were better for supramolecules than for the components in true-solution phase because of supramolecules was easily ingested by cells and targeted the brain and lungs, indicating that the supramolecules are key to the antipyretic mechanism of the Baihu decoction [40].

#### 3.1.2. Huanglian Jiedu Decoction

The Huanglian Jiedu decoction is composed of four commonly used medicinal herbs, namely, *Coptidis Rhizoma*, *Radix Scutellariae*, *Phellodendri Cortex*, and *Gardeniae Fructus*. There was obvious precipitation in the decoction, and the precipitation rate reached 7.13% [41]. Fang [42] used the HPLC method to determine the precipitation components of the Huanglian Jiedu decoction and found that 81% of the precipitation was organic acids and alkaloids, of which baicalin accounted for 42.12% and berberine accounted for 31.17%. This study revealed that the compound precipitation from the Huanglian Jiedu decoction was mainly composed of an acid–alkali complex. Baicalin is acidic due to the presence of carboxyl groups in its structure, and it is prone to precipitation reactions with alkaloids such as berberine. Therefore, from the perspective of molecular thermodynamics, all the sources of precipitation, parameters of interaction, and binding abilities of different medical combinations during the formation of the precipitate in the Huanglian Jiedu decoction were explored. The original decomposed-recipe experiment indicated that the combinations that could produce obvious precipitates when they were mixed were *Scutellariae Radix*–*Coptidis Rhizoma* and *Scutellariae Radix*–*Phellodendri Chinensis Cortex*. The amount of precipitation for the *Scutellariae Radix*–*Coptidis Rhizoma* was the highest. Then, isothermal titration calorimetry (ITC) was used to determine the binding heat and thermodynamic parameters of the binding reactions, and the results show that the precipitation-formation process is a chemical reaction that drives the non-covalent bonding of enthalpy, rather than a simple physical aggregation and adsorption precipitation. Therefore, it is believed that the precipitate in the Huanglian Jiedu decoction is formed by self-assembly [43]. The compositions of both the supernatant and naturally supramolecules of the Huanglian Jiedu decoction were further analyzed by UHPLC–Q–Orbitrap HRMS. The results showed that the compositions of the supernatant and the supramolecules were the same. Due to the self-assembly complexation, the supramolecule’s content of baicalin and berberine was significantly higher than that of the supernatant [44]. Based on these studies, Zhang [14] used baicalin and berberine to synthesize and form the precipitate in the Huanglian Jiedu decoction and found that baicalin and berberine formed complex molecules at a molar ratio of 1:1 through electrostatic attraction. From the basic unit, further assembly forms the supramolecules in the Huanglian Jiedu decoction. Cobalt chloride was used to induce PC12 cells to establish a nerve-injury model. The supramolecular precipitate in the Huanglian Jiedu decoction and the simulated synthetic baicalin–berberine supramolecules showed good protective effects.

#### 3.1.3. Gegen Qinlian Decoction

The Gegen Qinlian decoction is composed of four traditional Chinese medicines: *Pueraria lobata*, *Scutellaria baicalensis*, *Coptis chinensis*, and *Glycyrrhizae*. The changes in the content of active ingredients before and after the formulation of the Gegen Qinlian decoction was determined by HPLC. The results showed that, when *Pueraria lobata* was decoctioned with *Coptis chinensis* and *Scutellaria baicalensis*, respectively, it could solubilize berberine, palmatine, baicalein, and wogonin. Among them, the reason *Puerariae* promotes the dissolution of baicalein and wogonin may be the formation of molecular complexes [45]. Hu [21] found that puerarin, daidzein, and daidzein are the main components of supramolecules in the Gegen Qinlian decoction and have good activity in vitro. Guo [46] used ultrafiltration centrifuge tubes to filter the Gegen Qinlian decoction. After intercepting most of the supramolecule particles, the ingredients in the decoction were tested. The results showed that the contents of several main active ingredients in the decoction were reduced to varying degrees after ultrafiltration. This shows that the supramolecules in the decoction are an important substance that exerts medicinal effect. Experiments by Lin also proved this point. The supramolecules showed stronger activities than the supernatant on many tests. In vivo experiments showed that the supramolecules of the Gegen Qinlian decoction showed a stronger hypoglycemic effect. In vitro experiments showed that the supramolecules in the decoction had stronger antioxidant effects, better protective effects on cells, and were basically non-toxic. High absorption rates of baicalin indicated that the supramolecules changes pharmacokinetics of Gegen Qinlian decoction and improves the bioavailability of insoluble phytochemicals, like baicalin, may be essential for the synergistic actions in the herbal decoction. It showed that the supramolecules in the Gegen Qinlian decoction had a better medicinal effect [20]. When investigating the protein self-assembly behavior during the Gegen Qinlian decoction, Lin separated Pueraria protein and Coptis protein and found that the two proteins could form supramolecules under simulated decoction conditions. The experimental results showed that the efficiency of Pueraria protein encapsulated puerarin was 33.88%, and the efficiency of Coptis protein encapsulated berberine hydrochloride was 44.2% [47].

#### 3.1.4. Maxing Shigan Decoction

The Maxing Shigan decoction is a classic prescription consisting of *Ephedra*, *Radix Glycyrrhizae*, *Semen Armeniacae Amarum*, and *Gypsum*. Studies have found that the chemical components of the Maxing Shigan decoction intertwine to form a new physical phase during the decoction process, which leads to the heterogeneous distribution of the ingredients of the decoction. Pharmacodynamic experiments showed that the supramolecular structure of the Maxing Shigan decoction has good antibacterial activity, and the composition test showed that the supramolecules contain organic active small molecules such as ephedrine; amygdalin and glycyrrhizic acid; Ca, K, and Mg; and other inorganic ingredients [34]. Research by Zhou showed that ephedrine (99.7%) and pseudoephedrine (95.5%) in the Maxing Shigan decoction are mainly present in supramolecules. It is speculated that amphiphilic molecules, such as ephedrine and pseudoephedrine, are adsorbed on the supramolecules through hydrogen bonding, electrostatic interactions, and van der Waals attraction [22]. Du [48] compared the in vitro change in antiviral activity in the Maxing Shigan decoction, determined by the MTT method, before and after filtration with a 0.45 μm cellulose acetate film. The results showed that the antiviral activity of the decoction was significantly reduced after filtration. The reason is that a large number of supramolecules are removed by filtration, which proves that the anti-influenza-virus activity of the Maxing Shigan decoction is related to its supramolecules.

#### 3.1.5. Siwu Decoction

The Siwu decoction is composed of *Rehmannia glutinosa*, *Angelica sinensis*, *Paeonia lactiflor*a, and *Ligusticum chuanxiong*. Zhang used differential centrifugation to obtain supramolecules with a particle size distribution of 100–1000 nm in the Siwu Decoction. Assays showed that the supramolecules contained a large amount of protein and polysaccharides and a small amount of DNA. Pharmacological studies showed that heme synthesis, degradation, and protein binding were closely related with the supramolecules, and it had regeneration-promoting effects on the damage of hematopoietic function. Therefore, it is speculated that supramolecules greatly contribute to the medicinal effect of the Siwu Decoction [23].

#### 3.1.6. Shaoyao Gancao Decoction

The Shaoyao Gancao decoction is composed of two traditional Chinese medicines: *Paeoniae* Radix and *Glycyrrhizae* Radix. The main active components of the monarch medicine *Paeoniae* Radix are monoterpene glycosides. These glycosides are more polar and difficult to absorb in the gastrointestinal tract. However, when it is combined with *Glycyrrhizae* Radix, the absorption efficiency for the active ingredients of *Paeoniae* Radix is effectively improved [49]. Particle-size analysis and morphological observation showed that there were supramolecules with a particle size of approximately 200 nm in the Shaoyao Gancao decoction, and the supramolecules were irregularly spherical under TEM observation. The experimental results showed that the supramolecules in the Shaoyao Gancao decoction could effectively encapsulate the active ingredients in *Paeoniae* Radix. After entering the body, it not only exerts a sustained release effect but also improves the absorption efficiency for the drug in the ileum [24]. Shen prepared paeoniflorin-loaded glycyrrhizic acid supramolecules using the ultrasonic dispersion method to improve the oral absorption of paeoniflorin. The in vivo pharmacokinetics showed that the C_max_ and AUC0–t values of paeoniflorin encapsulated by supramolecules formed by glycyrrhizic acid were approximately 2.18 and 3.64 times higher than those of paeoniflorin in solution [50].

### 3.2. Research into the Supramolecules of Medicinal–Pair Decoction

#### 3.2.1. Aconiti Laterdis and Glycyrrhizae Radix Co-Decoction

*Aconiti Laterdis* and *Glycyrrhizae* Radix, a classic medicinal combination, can reduce the toxicity and increase the effects after compatibility, but the mechanism is not yet clear. In analyzing the differences in the physicochemical properties of the *Aconiti Laterdis* and *Glycyrrhizae* Radix decoction before and after combination, Chen found that the particle size of the combined decoction was larger than that of the single decoction. Therefore, it is speculated that the supramolecules produced by the combined decoction may be the material basis for synergism and detoxification after compatibility [51]. Then, they used HPLC–MS to identify 36 components from the supramolecules in the combined decoction, among which there were 11 compounds from *Glycyrrhizae* Radix and 25 compounds from *Aconiti Laterdis*. According to these confirmed compounds, the alkaloid compounds in the combined decoction were significantly different from those of the single decoction. This shows that there were some changes in the alkaloid compounds in the decoction after the combination of *Glycyrrhizae* Radix [52]. The contents of six ester alkaloids in the supramolecules formed before and after compatibility were compared simultaneously using the HPLC–TOF–MS method, and the supramolecular changes were identified using FTIR and second-derivative spectra. The results showed that, in the process of co-decocting, a large number of ester alkaloids in *Aconiti Laterdis* combined with the components in *Glycyrrhizae* Radix to form a supramolecule. It is speculated that the mechanism may be the association between the tertiary amine N in the alkaloid and the carboxyl in the glycyrrhizic acid [53]. Zhang [54] investigated the intestinal absorption and pharmacokinetic characteristics of the three diester-types diterpene alkaloids in the supramolecules of the co-decoction. The results showed that the diester-type diterpene in the supramolecules prevents dose dumping and prolongs the average residence time, thereby effectively reducing the toxicity of aconite after oral administration.

Apart from acid–base neutralization, studies have shown that glycyrrhizae protein is also an entry point to clarifying the mechanism of reducing toxicity. Rao [25,55] found that glycyrrhizae protein could be separated by anion-exchange chromatography. When the pH was 5, the glycyrrhizae protein could form supramolecules with a stable particle size with aconitine, and the embedding rate was 28.22%. Acute toxicity experiments in mice showed that glycyrrhizae protein attenuated the toxicity after embedding aconitine.

#### 3.2.2. *Glycyrrhizae* Radix–*Coptis* Chinensis Co-Decoction

*Glycyrrhizae* Radix–*Coptis* Chinensis has a wide range of clinical applications, but the drug pair produces a large amount of self-precipitation when co-decocted. When studying the supramolecules of the co-decocting of *Glycyrrhizae* Radix–*Coptis* Chinensis, it was found that the components in the supramolecules of the combined decoction were significantly different from those of the single decoctions, and the combined decoction contained more active ingredients [56]. Zhao [57] used HPLC to detect the components in the supramolecular precipitate; the results showed that it was mainly small molecules such as glycyrrhizic acid and berberine. The investigation of the solubility of supramolecular precipitates in digestive conditions showed that the supramolecules formed by the compatibility of *Glycyrrhizae* Radix and *Coptis* Chinensis allowed for the slow release of berberine. The formed supramolecules not only ensure the therapeutic effect of berberine but also prevent the side effects of *Coptis* Chinensis inhibiting yang, damaging the spleen and stomach, confirming the rationality of the compatibility law for *Glycyrrhizae* Radix and *Coptis* Chinensis. Li [26] found that *Glycyrrhizae* Radix crude protein and berberine could form spherical supramolecular particles under the induction of weak bonds, such as electrostatic attractions and hydrophobic interactions, after co-decoction. The antibacterial activity of the supramolecules is significantly better than that of berberine and mechanical mixtures. The amino acid residues on the surface of glycyrrhizae protein could make itself adsorbed on Staphylococcus aureus. Therefore, glycyrrhizae protein–berberine supramolecules may adhere to the bacterial surface after interacting with bacteria, and the supramolecules continuously release berberine, which increases the concentration of berberine around the bacteria and increases the uptake, resulting in a stronger antibacterial effect. These experiments indicating that the interaction between glycyrrhizae protein and berberine during the heating process promotes a better effect of the medicinal ingredients.

### 3.3. Research into the Supramolecules of a Single-Drug Decoction

#### 3.3.1. Banlangen Decoction

*Isatis indigotica* Fort is one of the few Chinese medicines with good antiviral effects. Lin [27] tracked and compared the changes in the components of *Isatis indigotica* before and after decoction and found that spherical polymers formed during the boiling process. The supramolecules were formed by the inherent natural components in the decoction, such as proteins, sugars, amino acids, and fatty acids, through self-assembly. The characterization results from TEM and laser particle size analysis showed that the supramolecules were composed of a series of particles of different sizes. Further research has shown that the pH of the buffer during the boiling of *Isatis indigotica* determines the pH response of the supramolecules, and this pH response is microscopically manifested as the aggregation and dispersion of the supramolecules. Among them, the Banlangen decoction shows the lowest light-scattering intensity when the pH is close to neutral, and the light-scattering intensity increases under acid and alkali conditions, indicating that the particles of supramolecules become larger. Experiments show that the supramolecules of different forms have different antiviral effects. He [58] used gel chromatography to separate and purify the supramolecules in the Banlangen decoction and found that this supramolecule shows not only pH responsiveness but also temperature responsiveness. Zhou [59] identified two constitutive glycosylated proteins from supramolecules in the Banlangen decoction. The N-terminal amino acid sequences are V–X–R–E–V–V–K–D–I and V–V–R–E–V–V–K–D–I–A–G–A–V–Q–T–N–E–Q–Y. In order to clarify the structure, cDNA cloning and glycosylation–site analysis were carried out. The primary-structure comparison showed that the two glycosylated proteins have high homology, representing allelic variants of the same gene. Based on this, they obtained the highly homologous amino acid sequence of the non-glycosylated protein. Furthermore, they used pepsin hydrolysis and MS to identify four possible glycosylation adducts in the supramolecules. From these studies, it could be observed that the supramolecules isolated from the Banlangen decoction were a smart nanocomponent composed of a boiling-stable protein, which was pH responsive and temperature responsive, and could be used as a prototype in the future to develop a smart, safe, and stable drug-delivery vehicle.

#### 3.3.2. Taizishen Decoction

Cai [28] separated the supramolecules in the Taizishen decoction by gel-filtration chromatography and analyzed their immune activity. They found that the isolated supramolecules could stimulate the proliferation of mouse spleen cells and promote the secretion of the immune factors IL–10, IL–13, TNF–α, and IFN–γ. This proved that the supermolecule is the main medicinal ingredient of the Taizishen decoction. Weng [60] obtained the crude protein from the Taizishen decoction using ammonium sulfate sedimentation technology. After heating at 100 °C for 30 min and adjusting the pH to 5.70, supramolecules with a particle size of approximately 70 nm were obtained, which could effectively improve the solubility of curcumin.

## 4. Mechanism of Formation of Supramolecules in Traditional Chinese Medicine Decoction

### 4.1. Alkaloids and Acidic–Form Supramolecules

Acid–base neutralization is a common reaction in the formation of supramolecules in traditional Chinese medicine decoctions. When herbs containing acidic components are decocted with the herbs containing alkaloids, the acidic components and alkaline dissolved in the decoction undergo a neutralization reaction. Due to the large relative molecular mass of the formed supramolecule complex, it easily precipitates. The alkaline components in traditional Chinese medicine are mainly alkaloids, such as berberine, aconitine, and ephedrine. There are many types of acidic components, such as organic acids, glycosides, and tannins.

The molecular structure of organic acids contains carboxyl groups, so they can react with alkaloids. When studying the drug pair of *Coptis* Chinensis and *Polygala*, it was found that the effective ingredients, berberine and 3,4,5–methoxycinnamic acid, could form supramolecules through hydrogen bonding and π–π stacking interactions. The efficacy experiments showed that the supramolecules could specifically bind on the surface of drug-resistant *Staphylococcus aureus*, which is superior to the clinical antibacterial drugs amoxicillin and norfloxacin [29].

**Figure 4:**
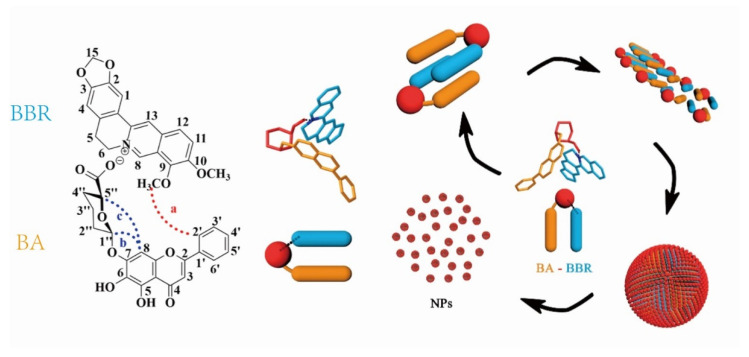
Schematic representation of the self-assembly mechanism of the berberine (BBR) and baicalin (BA) [61].

Most glycosides are neutral or acidic, meaning that they can easily react with alkaloids by acid–base neutralization to generate supramolecular precipitates. For example, baicalin is a typical acidic glycoside due to the presence of a carboxyl group in its structure. Thus, the Huanglian Jiedu decoction is a representative formula that forms supramolecules by acid–base neutralization between glycosides and alkaloids. Inspired by the Huanglian Jiedu decoction, Li [61] used baicalin and berberine to simulate synthesized supramolecular precipitates. Baicalin (BA) and berberine (BBR) first formed one-dimensional complex units, which the combination occurred at the −COO− of baicalin and N^+^ of berberine driven by electrostatic interaction. Subsequently hydrophobic interaction drove further three-dimensional self-assembly: the hydrophilic glucuronic acid toward the outside and the hydrophobic parent nucleus toward the inside, as shown in Figure 4. Pharmacological experiments showed that the supramolecules not only have good biocompatibility but also have significantly better antibacterial activity than berberine. With the hydrophilic glucuronic acid toward the outside, the supramolecules exhibited stronger affinity to bacteria, thereby inducing the collapse of the bacterial population and a decrease in biofilm. In addition, studies have found that the baicalin–berberine supramolecules not only have a certain regulatory effect on glucose uptake but also showed a good therapeutic effect in visceral allergy and diarrhea in IBS–D model mice [62,63].

Tannins are a group of polyphenolic compounds with a complex structure. More than 70% of Chinese medicines contain tannins. When researching the compatibility of alkaloids and tannins, it was found that the alkaloids in *Corydalis* could easily interact with the tannins in *Burnet*, *Polygonum cuspidatum*, *Betel nut*, or *Sophora japonica* to form insoluble supramolecular precipitates. The supramolecules reduce the dissolution rates for active ingredients and extend the time for which the active ingredients exert their effects in the body [64]. The formation of supramolecular structures through alkaloids and tannins may an effective way to prepare sustained-release preparations.

### 4.2. Self-Assembly of Proteins

Proteins are biological macromolecules that have shapes that are approximately spherical. Proteins have good solubility in water because their hydrophobic amino acid residues are generally located inside the structure and their hydrophilic amino acid residues are outside. Between the molecules of the protein or proteins and other molecules will form supramolecular microspheres due to specific recognition and binding abilities, such as electrostatic attractions, van der Waals forces, hydrophobic forces, and hydrogen bonding. The Angelica sinensis protein, which was isolated from *Angelica sinensis*, can self-assemble to form supramolecules with a uniform particle size after heating at 100 °C for 15 min with a pH of 8.0. Some changes occur during the heating process, including a shift in the ANS fluorescence wavelength, decrease in the ANS fluorescence intensity, decrease in the α–helix content, and increase in the β–sheet content. This phenomenon indicates that the Angelica sinensis protein structures unfolded and the hydrophobic groups were exposed and aggregated, thereby forming supramolecules. Compared with the isolated Angelica sinensis protein, the heated protein formed supramolecular structures, improved antioxidant capacity, and more importantly, possessed the ability to encapsulate drugs. Further research found that supramolecules of Angelica sinensis protein have the ability to load the insoluble drug ferulic acid. The embedding rate for ferulic acid was approximately 30%, and the drug-loading rate for the supramolecules was approximately 65%. Furthermore, the supramolecules had various biological activities and high safety [30]. This study showed that the supramolecules of Angelica sinensis protein represent a new type of nanocarrier with a simple preparation method, capable of embedding drugs and showing biological safety. Weng [65] simulated the process of decoction to construct Radix pseudostellariae protein supramolecules and tried to embed curcumin. Curcumin could combine with Radix pseudostellariae protein through hydrophobic interactions and quench the intrinsic fluorescence of the Radix pseudostellariae protein. The curcumin in the supramolecules exhibited good thermal stability and light stability. The supramolecules had stronger reducing power than free curcumin, showing an additive effect between curcumin and Radix pseudostellariae protein.

It is obvious that the supramolecules formed by the proteins of traditional Chinese medicine can not only serve as drug carriers to embed poorly soluble drugs, but can also exert synergistic effects, increasing the efficacy of drugs. The protein supramolecules with high biocompatibility obtained from traditional Chinese medicine broaden the application fields for protein components. They can not only provide a basis for the preparation of self-assembled nanoparticles of the active ingredients of traditional Chinese medicine and the study of synergistic effects but also provide new ideas for the design of poorly soluble drug carriers with a new direction.

### 4.3. Self-Assembly of Polysaccharides

The abundant hydrophilic groups in the carbohydrate skeleton structure enable polysaccharides and saponin to spontaneously aggregate with other structural units through non-covalent bonds under specific conditions to form various forms of supramolecules [66]. Both the helical structure of high saccharides and the closed-loop polymerization structure of oligosaccharides can form supramolecules that embed small molecules. At the same time, carbohydrates are good hydrogen donors and acceptors, which can interact with other molecules to form supramolecular systems. Zhao [67] isolated and obtained the acidic branched polysaccharide of vinegar-baked *Radix bupleuri*. The study found that the polysaccharide not only has high solubility for baicalin and rhein, much better than that of Tween 80, but also significantly increased the blood concentrations of baicalin and rhein in vivo. The solubilization mechanism might be the self-assembly of the acidic branched polysaccharide to form micelle-like aggregates in water, which can encapsulate water-insoluble constituents through the interaction of both hydrogen bonding and hydrophobic forces, thereby increasing the solubility and blood concentration. Ginsenoside Ro is a natural anionic biosurfactant derived from Ginseng. Studies showed that ginsenoside Ro can form vesicles via the closure of oblate membranes. At low concentrations, saikosaponin a is solubilized in the palisade layer of the ginsenoside Ro vesicles. At high concentrations, saikosaponin a interacts with ginsenoside Ro molecules to form mixed vesicles with ginsenoside Ro adsorbing on the surfaces of the vesicles, thereby significantly increasing the solubility of saikosaponin a [68]. Platycodin, a type of plant-based biosurfactant, has been confirmed to have the potential to enhance the solubility of hydrophobic drugs and function as a drug carrier. Studies have revealed that a rich variety of aggregate morphologies appear with changes in the structure or the concentration of saponins including spherical, ellipse, and oblate micelles and vesicles; multilamellar vesicles; multicompartment vesicles; and tubular and necklace-like micelle [69]. Ding [70] found that combining menthol and platycodin D, the main active ingredients in *Peppermint* and *Platycodon grandiflorum*, can increase the solubility of the menthol by forming supramolecules and achieving the effect of compatibility and synergy. These studies have shown that the supramolecules formed by polysaccharides and saponins have better effects on improving the solubility of drugs and that most of the components have better biocompatibility and the potential to be developed as special drug carriers.

### 4.4. Self-Assembly of Triterpenoids

Triterpenoids are terpenoids that have a basic skeleton that consists of 30 carbon atoms. The structural characteristics of the rigid framework, flexible alkyl side-chains, and multi-chiral centers of triterpenoids make it easy to form a self-assembly structure due to the influence of non-covalent bonds such as hydrophobic interactions, hydrogen bonding interactions, and π–π stacking interactions [71]. Glycyrrhizic acid is a triterpenoid with a high content of *Glycyrrhizae* Radix. It is composed of one molecule of hydrophobic glycyrrhetinic acid and two molecules of hydrophilic glucuronic acid. Glycyrrhizic acid can self-assemble in water to form a right-handed long-fiber supramolecular structure with a hydrophobic inner cavity. As the concentration increases, it forms a supramolecular hydrogel with a network structure [72]. Liu’s research found that glycyrrhizic acid had significant solubilizing and stabilizing effects on flavonoids. These effects are negatively correlated with the solubility and stability of the flavonoids themselves. Baicalein was selected as the model drug. The encapsulation efficiency of the supramolecules prepared under the optimal conditions was approximately 80%, the drug loading was approximately 9%, and the solubility of baicalein increased from 0.15 to 690.90 μg/mL^–1^ [73]. Yang [74] studied the compatibility of *Glycyrrhizae* Radix and found that the glycyrrhizic acid in *Glycyrrhizae* Radix can encapsulate the insoluble active ingredients puerarin and baicalin in the supramolecular structure, thereby increasing the solubility of the drug and improving the bioavailability. You [75] prepared the glycyrrhizic acid-baicalein supramolecular structure. In vitro drug-release experiments showed that the cumulative drug release of baicalein in pH 6.8 and pH 8.3 buffer after 6 h was 18.20% and 47.96%, respectively, indicating that the supramolecules had a slow-release effect on baicalein and that the release rate can be adjusted by changing the pH. The supramolecular structure formed by glycyrrhizic acid can not only increase the solubility of poorly soluble drugs and improve the stability of the encapsulated drugs but can also control the release. In the future, scientists can look for compounds with similar structures to glycyrrhizic acid so as to discover more traditional Chinese medicinal ingredients with good carrier characteristics.

### 4.5. Decoctosome

Exosomes are nano-sized vesicle-like bodies that are actively secreted by cells and can effectively deliver their molecular cargo to recipient cells. A class of exosome-like supramolecules has been isolated and identified from a decoction of traditional Chinese medicine, named the decoctosome. The decoctosome contains lipids, proteins, RNA, and other components, which may be the main components of traditional Chinese medicine. When studying the Dandelion decoctosome, Li [76] used specific chemical inhibitors of the endocytic pathway and the method of knocking out key proteins in the endocytic pathway to evaluate the mechanism by which the fluorescently labeled decoctosome entered the cell. The results showed that pro-lysosomal substances can significantly inhibit the entry of the decoctosome to the cells, and the giant pinocytosis inhibitors cytochalasin D and amiloride can significantly reduce the intracellular uptake of the decoctosome in a dose-dependent manner. Knocking down Rac1 and PAK1 also effectively inhibited decoctosome entry into cells. These results prove that the Dandelion decoctosome enters A549 cells through endocytosis, mainly mediated by giant pinocytosis, providing new ideas for the mechanism of Chinese medicinal supramolecular structures in delivering drugs.

### 4.6. Others

Du [77] found that two lipids extracted from Rhodiola decoction could form supramolecular structures with Rhodiola mRNA, and the supramolecules could promote the entry of mRNA to human lung and gastrointestinal cells to exert its effects. Liu [78] found that the supramolecular structure formed by the coordination and assembly of luteolin and ferric ions not only enhanced stability and solubility but also introduced a supramolecular photothermal effect. The formed supramolecules can be used as either chemotherapeutic agents or photothermal agents, with a wide range of application prospects. 

## 5. The Influence of the Supramolecular Structure on Drug Efficacy

Summarizing the research on the supramolecular structures of traditional Chinese medicine decoctions, it can be observed that various components in traditional Chinese medicine self-assemble into supramolecular systems under the induction of weak bonds. Studies have shown that the compositions of supramolecules are consistent with the main components in the decoctions [79], suggesting that the supramolecular structure in a decoction contains many active ingredients, which may have biological activities and are worthy of investigation. Increasing research shows that, compared with free monomer components, the biological activity of supramolecules is significantly improved. 

There are two main reasons for this. The first reason is that the components in the supramolecular structure have a synergistic effect. For example, the antibacterial effect of the supramolecular structure formed by berberine and rhein is significantly better than that for each component individually. The study found that this synergistic effect is due to the fact that supramolecules can adhere to the surface of bacteria, whereas monomers cannot, thereby increasing the concentration of the drug around *Staphylococcus aureus*, resulting in a good bactericidal effect [17]. The second reason is that the formation of a supramolecular structure increases the solubility of insoluble components and prolongs the time to take effect in vivo. A supramolecular structure composed of 93.7% protein and 6.3% sugar was isolated from *Alisma orientale*. This supramolecule can encapsulate 2,3–acetyl alismanol B, which is insoluble in water, and effectively improves the efficacy of the drug [80]. Summarizing these studies, it can be observed that the supramolecular structure in the decoction plays an important role in the process of exerting medicinal effects. This may be a new perspective for explaining the material basis of the medicinal effects of traditional Chinese medicine. It also proves that it is an unscientific view to removing the supramolecular structure in the decoction in order to pursue the clarification of the medicine during the preparation process.

## 6. Research Significance of the Supramolecular Structure

### 6.1. Combination of Supramolecular Chemistry and Basic Theories of Traditional Chinese Medicine

The compatibility of traditional Chinese medicine is based on the guidance of the theory of traditional Chinese medicine according to the needs of curing disease and the characteristics of the medicine, and then selectively using a combination of two or more medicines to reduce the toxicity and increase the efficiency. Pharmacodynamic experiments have proved the authenticity of compatibility reducing toxicity, but opinions on the mechanisms vary. The current chemical research on compatibility reducing toxicity mainly focuses on the recognized structural changes of toxic components, while ignoring the fact that the toxic components may form supramolecular structures with other components during the compatibility process.

**Figure 5:**
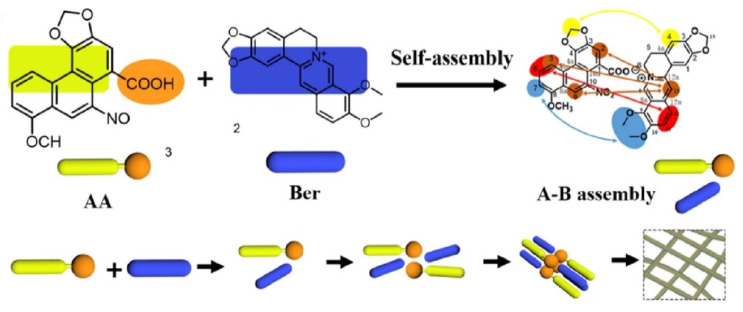
Schematic representation of the self-assembly mechanism of the berberine (Ber)-aristolochic acid (AA) supramolecular structure [31].

When Wang studied the compatibility of *Coptidis Rhizoma* and *Aristolochia debilis*, they found that the active ingredients, berberine (Ber) and aristolochic acid (AA), formed linear heterogeneous supramolecules through electrostatic attraction and π–π stacking, with the hydrophobic groups outside and the hydrophilic groups inside during the drug combination practice, as shown in Figure 5. This structure blocks the toxic site of aristolochic acid and hinders its metabolism. Therefore, the supramolecules significantly reduce the toxicity of aristolochic acid and alleviate the acute kidney injury caused by it [31].

The glycyrrhizic acid in *Glycyrrhizae* Radix can precipitate with the alkaloids in *Strychnos nux–vomica*, and the toxic substances was significantly reduced after they were combined. Therefore, it is speculated that the reason for the reduced toxicity of *Strychnos nux–vomica* when combined with *Glycyrrhizae* Radix is related to the supermolecules produced [81,82]. These studies suggest that scientists should pay attention to the supramolecular structure produced during the compatibility process. Toxic substances and detoxification components form supramolecules, reducing toxicity, which may be one of the theories explaining compatibility.

### 6.2. The Guiding Role of Supramolecular Structures for Clinical Preparations

As the supramolecular structure in the decoction has a high surface free energy, it will spontaneously aggregate and precipitate, thus forming what people think of as the “precipitate” in the decoction. The particle size of the nanophase in the Liujunzi decoction is concentrated at 100 nm, and there are a small number of particles larger than 100 nm, indicating that different degrees of agglomeration occur in the mixed system [83]. The theory of traditional Chinese medicine states that the precipitate should be drunk together with the decoction to achieve the therapeutic effect. As early as the 1980s, studies found that the decoction was in a suspended state after being decocted, and the curative effect of the decoction would be reduced after the precipitate was removed [84]. However, in the process of the extraction and production of the decoction by pharmaceutical companies, the precipitate in the decoction is mostly filtered out and discarded together with the dregs of the decoction, which also affects the efficacy of the drug [85]. Wan [86] studied the dispersion properties of 22 traditional Chinese medicine decoctions and found that there were a large number of nanoscale supramolecular structures in the decoctions after centrifugation, which may be composed of proteins, polysaccharides, and surfactants. Later, they used *Salvia miltiorrhiza* as a model medicinal and found that, after centrifugation, alcohol precipitation, flocculation, salting out, and other impurity-removal methods, the nanoparticles in the decoction were significantly reduced, and both water-soluble and fat-soluble active ingredients were lost. Many studies have shown that the supramolecules in the decoction may encapsulate or adsorb the effective ingredients in the liquid. Therefore, the blind pursuit of clear solutions in the preparation process not only is the reason for the inconsistent clinical efficacy, but also leads to the unintentional waste of medicine.

At present, the granules obtained by single-drug decoction are often used clinically for compound compatibility, but the scientific validity of this approach remains to be verified. Li found that the sphingosine and sRNA separated from the *Rhodiola* decoction can form supramolecules after heating. The heating process promotes the assembly of sRNA and sphingosine to form a stable state, resulting in a good therapeutic effect [87]. A large number of flocculent precipitates are produced in the Sanhuang Xiexin decoction [88]. From the perspective of the phase state of the decoction, it can be found that the reaction and combination degree of the active ingredients of the combined decoction of the Sanhuang Xiexin decoction and the single decoction were quite different. Based on this, it is speculated that, during the decoction process, the medicinal substance reacts sufficiently to form a more stable supramolecular structure with a smaller particle size [89]. Pharmacological experiments showed that the analgesic, antipyretic, and anti-inflammatory effects of the Sanhuang Xiexin decoction were better than those of a combined single decoction [90]. When the classic medicines *Coptidis Rhizoma* and *Evodia* are co-decocted, the alkaloids in *Coptidis Rhizoma* and the flavonoids in *Evodia* form supramolecules that are insoluble. However, if the granules made from single herbal decoctions are used together without the combined decoction process, the curative effect is not as good as that of the combined decoction [91]. It is not difficult to observe from these studies that co-decoction is one of the main conditions for the generation of supramolecular structures in decoctions. Therefore, the production characteristics for supramolecules may also be one of the reasons the granules of the single medicinal decoction are less effective than those of the combined decoction. In this case, research on the supermolecules formed during the decoction of traditional Chinese medicine can not only clarify the material basis of the drug’s therapeutic effect but also provide certain guidance for clinical preparations.

## 7. Identifying the Current Research Gap and Prospective Research Direction

Previous studies have shown that supramolecular structures were an important class of pharmacodynamic substances in decoction. Based on this basic research, the biosafety of supramolecules is the first aspect that should be paid attention to when further transforming into practical applications. To confirm the biosafety of berberine-cinnamic acid supramolecules, an in vitro hemolysis assay, cytotoxicity test, and in vivo zebrafish model were carried out. Supramolecules showed no obvious cytotoxic effects on a variety of cells. The result of the hemolysis test showed that there was no obvious hemolysis in rats’ red blood cells treated with supramolecules. Therefore, supramolecules had good biocompatibility and were suitable for blood contact applications. In addition, the result of the zebrafish assay indicated that supramolecules had little influence on zebrafish larvae growth. The available data suggested that berberine–cinnamic acid supramolecules had good biocompatibility. Similarly, Angelica sinensis protein promoted the proliferation of human normal hepatic cells and would not induce the hemolysis of healthy red blood cells, which also has biosafety. At present, the research on the biosafety of the supramolecular structure in decoction is mainly based on cytotoxicity experiments. In the future, it can be further expanded by referring to the research of nanomedicines: to carry out systematic toxicological studies in animal models to evaluate the toxicity of supramolecular structures at different levels (short-term and long-term toxicity, neurotoxicity, genotoxicity, immunotoxicity), and to discover the toxic effects and toxic target organs of supramolecules, providing evidence for possible clinical adverse reactions.

After the supramolecular structures entered the body, the pharmacokinetic behavior mainly depended on the properties of the structures. The particle size of supramolecules not only affects drug loading and drug releasing, but also, was closely related to drug delivery mechanisms, pharmacokinetics, biodistribution, and clearance pathways. The structure and shape of supramolecules affect the interactions with proteins and cell membranes. The surface charge of supramolecules affects their aggregation properties, stability, and biodistribution. Supramolecules may be absorbed via the transcellular pathway through epithelial cells that line the intestinal wall or through specialized microvilli cells that cover lymphoid follicles [92]. The intestinal absorption of baicalin from Gegen Qinlian decoction was assessed in a Caco-2 cell monolayer model for evaluating whether formation of supramolecules altered the bioavailability of Gegen Qinlian decoction. Results showed supermolecular complexes change the pharmacokinetics of Gegen Qinlian decoction and improve the bioavailability of insoluble phytochemicals. In addition, some studies have also proven that supramolecular structures can increase the bioavailability of drugs, but there are few studies on the pharmacokinetics of the supramolecular structure in the body, which should be regarded as a future research direction. On the other hand, an experiment on the enzyme stability of supramolecular structures has not been studied, and it can also be used as a future research direction for targeted therapy.

Stability also is a key indicator to evaluate whether a drug can potentially be used in a clinical application. The rhein hydrogel maintained good stability after being stored at room temperature for 3 months. Angelica sinensis proteins are suitable for short-term preservation at 4 °C and long-term storage at −20 °C radical scavenging. However, the results for the stability of supramolecules in Baihu decoction showed that aggregated particles began to appear at 8 h. Over time, the aggregation increased. This phenomenon may be caused by the supramolecules continuing to aggregate because of their small surface potential. The stability of some self-assembled products is still poor, and the preparation process is easily affected by factors such as external temperature and pH value. Many self-assembled products can only be stored for a few days at room temperature. Although low temperature conditions are conducive to their storage, this is a new challenge for production and use.

## 8. Application Prospects of Supramolecular Structure

With the deepening of research, scientists have discovered that the components can be assembled into supramolecular structures with specific structures and functions in a certain way based on the principle of self-assembly, using intermolecular forces as tools. Therefore, supramolecular chemistry has shown broad application prospects in the fields of food, industry, agriculture, medicine, and aerospace science.

Drug-delivery systems are the focus of modern pharmacy research, and self-assembly technology can also provide new ideas for the development of drug-delivery systems. The ideal natural drug carrier should be a self-assembled supramolecular structure formed without structural modification, with superior solubility, excellent therapeutic effects, and good biocompatibility. Traditional Chinese medicinal supermolecules have such unique drug-carrier characteristics. Pharmacological experiments have demonstrated that the supramolecular nanoaggregates in Baihu Tang are more easily absorbed by cells, and the fluorescein isothiocyanate signal of the nanoparticles is more abundant in the lung and brain than in other organs over time [93]. This study suggested that traditional Chinese medicinal supermolecules exhibit the characteristics of drug carriers that have good targeting. However, current research on the characteristics of decoction supramolecular drug carriers mainly focuses on loading the insoluble ingredients and increasing the solubility. Osamu Tanaka [94,95] found that the active ingredients in *Glycyrrhizae* Radix can significantly increase the solubility of saikosaponin a and saikosaponin b1. Zhou [32] found that, when simulating the decoction state, glycyrrhizae protein can form supramolecular nanoparticles with a stable particle size and can load approximately 51% of astragaloside IV. Rao [33] studied the supramolecular structure formed by the astragalus protein and found that, when the pH was 8.3 and the mixture was heated at 80 °C for 60 min, the supramolecular formation was stable, and the particle-size distribution was uniform. A supramolecular structure loaded with astragaloside IV was prepared, and the loading efficiency was 35.3% according to HPLC measurements.

These studies have clearly demonstrated that Chinese medicinal supermolecules have good drug-carrier properties, which can not only increase the solubility of insoluble drugs and improve the stability of encapsulated drugs but also produce targeting properties and control release. More importantly, different from chemical drugs, the characteristics of Chinese medicine make the supramolecules have good biocompatibility and safety for clinical application. To date, a variety of supramolecules have been used as drug carriers in experiments. It is hoped that, in the future, more traditional Chinese medicine supramolecular structures will be able to be used as clinical drugs to create better therapeutic effects.

## 9. Summary

Chinese medicine is one of the treasures of the Chinese nation, but the current research on Chinese medicine mainly focuses on the specific components of secondary metabolites, such as the medicinal effects of flavonoids, saponins, and proteins, while ignoring the interactions among the components. Many studies have shown that the precipitate in a decoction is actually a spontaneously formed supramolecular structure. However, most of the supermolecules formed by self-assembly have poor stability, which is why decoctions easily form precipitates. In the process of preparation, supermolecules are extremely susceptible to the external temperature, pH, and other factors, and many supramolecular structures can only be stored for a few days under normal temperature conditions. Although low-temperature conditions are beneficial for their storage, still is a challenge for production and use.

The research on the supramolecular structures in traditional Chinese medicine decoctions remains in its infancy, and the research methods are very limited; however, scientists should note that the concept of supramolecular chemistry has been formally put forward for more than 30 years. Supramolecular chemistry has been applied in other disciplines, and its mature research methods and ideas can be used for reference in the research of Chinese medicinal supermolecules. Therefore, although the study of self-assembled supramolecules in the field of Chinese medicine is relatively late, through the research of supramolecules and self-assembly in other fields, Chinese medicinal supermolecules will inevitably develop rapidly.

A decoction is the most important applicable form of traditional Chinese medicine. With the gradual discovery of supramolecular structures, research can not only reveal the scientific nature of traditional Chinese medicinal compounds but also solve the challenges encountered in the modern research process for traditional Chinese medicine. Research into the active components of traditional Chinese medicine and their mechanisms of action in the body provides new research ideas and methods. It is believed that, with the continued in-depth exploration of the supramolecular structures of traditional Chinese medicine, a wider range of pharmacological effects of traditional Chinese medicine will be revealed, and traditional Chinese medicine will attract increasing attention around the world.

## Figures and Tables

**Table 1:** Supramolecules in traditional Chinese medicine decoction and their characteristic.

Source	Biophysical	Biochemical	Therapeutic
co-decoction of Coptidis rhizoma and Cinnamomi cortex [15]	spherical particles; average particle size: 66 nm; zeta potential: −32.1 mV	ingredients: berberine and cinnamic acid; characteristics: pH sensitive, well biocompatibility	bacteriostatic activity
Huanglian Jiedu decoction [14]	spherical particles; average particle size: 174 nm;	ingredients: baicalin (42.12%), berberine (31.17%), coptis (5.89%), wogonoside (1.50%), palmatine (0.60%)	neuroprotective activity; regulate glucose uptake; good therapeutic effect in visceral allergy and diarrhea
*Rheum palmatum* decoction [16]	nanofibers; average diameter: 30 nm; length: several micrometers;	ingredients: rhein; characteristics: thixotropy; thermo-reversibility	neuroinflammatory prevention activity
co-decoction of *Coptis chinensis* and *Rheum palmatum* [17]	spherical particles; average particle size: 174 nm;	ingredients: rhein and berberine	antibacterial effect
*Puerariae thomsonii* Radix Aqueous decoction [18]	spherical particles; average particle size: 120 nm (0.07 g/mL); 185 nm (0.1 g/mL)	ingredients: Puerarin starch	ameliorate the hemorheological parameters
Baihu decoction [19]	spherical particles; average particle size: 100 nm; zeta potential: −3.11 mV	ingredients: new mangiferin, mangiferin, glycyrrhizin, ammonium, glycyrrhizin, polysaccharides, starches, inorganic elements, such as Si, Na, Ca, Mn, Fe, Cu, Fe; characteristics: selective organ targe	antipyretic effect; decrease inflammatory factors levels
Gegen Qinlian Decoction [20,21]	spherical particles; average particle size: 530 nm;	ingredients: Pueraria protein and Coptis protein, baicalin, berberine, puerarin, glycyrrhizic acid, and liquiritin	hypoglycemic and antioxidant activities
Maxing Shigan Decoction [22]	spherical particles; average particle size: 138 nm;	ingredients: ephedrine, pseudoephedrine	anti-influenza-virus activity
Siwu Decoction [23]	particle size distribution: 100–1000 nm	ingredients: protein, polysaccharides, DNA	regeneration-promoting effects on the damage of hematopoietic function
Shaoyao Gancao Decoction [24]	irregularly spherical particles; average particle size: 200 nm;	ingredients: glycyrrhizic acid, paeoni-florin	
*Aconiti Laterdis* and *Glycyrrhizae* Radix Co-Decoction [25]	average particle size: 238 nm;	ingredients: glycyrrhizae protein, aconitine	eliminates toxicity
*Glycyrrhizae* Radix–*Coptis* Chinensis Co-Decoction [26]	spherical particles; average particle size: 186 nm; zeta potential: −11.1 mV	ingredients: mainly small molecules such as glycyrrhizic acid and berberin	antibacterial effect
Banlangen Decoction [27]	particle size distribution: 50–500 nm	ingredients: proteins, sugars, amino acids, and fatty acids; characteristics: pH responsiveness, temperature responsiveness	antiviral effects
Taizishen Decoction [28]	average particle size: 70 nm;	ingredients: protein	immunomodulatory
Coptis Chinensis and Polygala Co-Decoction [29]	spherical particles; average particle size: 93 nm; zeta potential: −31.6 mV	ingredients: 3,4,5-methoxycinnamic acid, berberine	antibacterial activity
*Angelica sinensis* [30]	irregularly spherical particles; average particle size: 130 nm;	ingredients: Angelica sinensis protein	radical scavenging effects
*Coptidis Rhizoma* and *Aristolochia debilis* Co-Decoction [31]	cross-linked network structure; diameter: 50–200 nm; length: tens of micrometers; zeta potential: −38.5 mV	ingredients: berberine, aristolochic acid	reduce the toxicity of aris-tolochic acid
*Glycyrrhizae* Radix [32]	spherical particles; average particle size: 74 nm; zeta potential: −24.3 mV	ingredients: glycyrrhizae protein, glycyrrhizic acid	
*Astragalus* [33]	spherical or oval particles; average particle size: 151 nm; zeta potential: −26 mV	ingredients: astragalus protein	

## Data Availability

Not applicable.
